# The Relationship between Birth Weight and the Risk of Overweight and Obesity among Chinese Children and Adolescents Aged 7–17 Years

**DOI:** 10.3390/nu16050715

**Published:** 2024-02-29

**Authors:** Jiaqi Shi, Qiya Guo, Hongyun Fang, Xue Cheng, Lahong Ju, Xiaoqi Wei, Liyun Zhao, Qiuye Cao, Xiaolin Yuan, Li He

**Affiliations:** National Institute for Nutrition and Health, Chinese Center for Disease Control and Prevention, Beijing 100050, China; sjq8033@163.com (J.S.); guoqy@ninh.chinacdc.cn (Q.G.); fanghy@ninh.chinacdc.cn (H.F.); chengxue@ninh.chinacdc.cn (X.C.); julh@ninh.chinacdc.cn (L.J.); xq437073568@163.com (X.W.); zhaoly@ninh.chinacdc.cn (L.Z.); caoqy@ninh.chinacdc.cn (Q.C.); yuanxl@ninh.chinacdc.cn (X.Y.)

**Keywords:** birth weight, overweight, obesity, children, China

## Abstract

Obesity is a major public health issue in children and adolescents. Our study aimed to examine the impacts of birth weight on overweight and obesity among Chinese children and adolescents. Using data from the China National Nutrition and Health Surveillance of Children and Lactating Mothers in 2016–2017, we included 10,041 participants aged 7–17 years. According to birth weight, participants were categorized into six groups, and the birth weight category of 3000 to 3499 g was chosen as the reference group, containing the largest number of children. Logistic regression analyses were used to investigate the association of birth weight with the risk of being obese at 7 to 17 years of age in multivariable-adjusted models. A restricted cubic spline was utilized to show the odds ratios (ORs) of obesity at different birth weight levels. The adjusted ORs for overweight were 0.98 (95%CI 0.63, 1.53), 1.02 (95%CI 0.84, 1.25), 1.34 (95%CI 1.16, 1.55), 1.72 (95%CI 1.35, 2.18), and 1.17 (95%CI 0.71, 1.96) in several birth weight groups, compared with group C (3000–3499 g). The adjusted ORs for obesity were 0.82 (95%CI 0.48, 1.40), 0.77 (95%CI 0.60, 0.98), 1.33 (95%CI 1.13, 1.57), 1.97 (95%CI 1.53, 2.53), and 2.01 (95%CI 1.27, 3.19). Furthermore, children in the post-pubertal stage had a slightly higher risk of overweight and obesity than those in the pre-pubertal and pubertal stage. Moreover, these associations were stronger among boys. The lower part of normal birth weight range is associated with a lower risk of overweight and obesity in children and adolescents. However, higher levels of birth weight increase risk.

## 1. Introduction

Childhood overweight and obesity may currently represent one of the most severe public health concerns. Globally, over 340 million children and adolescents aged 5 to 19 were obese or overweight in 2016. Overweight and obesity rates among children and adolescents aged 5 to 19 have risen dramatically from just 4% in 1975 to just over 18% in 2016 [1]. In China, overweight and obesity rates in children and adolescents aged 6 to 17 have reached 19.0% (11·1% for overweight and 7·9% for obesity) [2]. These alarming numbers will continue to climb [3,4,5]. The World Obesity Atlas 2023 predicts that the prevalence of obesity is projected to rise fastest among children and adolescents, from 10% to 20% for boys and from 8% to 18% for girls, worldwide, between 2020 and 2035 [6].

Overweight and obesity are defined as abnormal or excessive fat accumulation that may impair health [1]. Childhood obesity not only affects physical and mental health but also poses serious long-term health risks [7,8]. Childhood obesity is associated with a higher chance of obesity, which can lead to hypertension, type 2 diabetes, coronary heart disease, and cerebral thrombosis in adulthood, seriously affecting the quality of life and increasing the disease and economic burden [8,9,10,11]. Therefore, the Children and Adolescents Obesity Prevention and Control Implementation Program proposes that the average annual increase in overweight and obesity rates from 2002 to 2017 be taken as the baseline, and the average annual increase in overweight and obesity rates among children and adolescents aged 0–18 years from 2020–2030 be reduced by 70% based on the baseline to achieve zero growth in overweight and obesity among children and adolescents in China [12]. The importance attached to the identification of potential risk factors is an issue of great significance.

Overweight and obesity are multifactorial diseases due to environmental, psycho-social, and genetic factors [6,13]. Nutritional status during fetal life is also one of the important factors. According to the developmental origins of health and disease hypothesis, an inadequate prenatal environment during fetal life is more prone to obesity and related outcomes later in life [14]. In this context, birth weight could be one key reaction to the nutrition status of fetuses, and a large number of studies have demonstrated that the influence of birth weight could persist into later life [15,16,17,18,19,20]. For instance, birth weight might be directly related to body mass index (BMI), which is known to be strongly associated with health outcomes [16,21]. Some studies have verified the relationship between birth weight and overweight or obesity. For example, two systematic reviews showed that a low birth weight (<2500 g) was associated with a decreased risk of obesity, while a high birth weight (>4000 g) was associated with an increased obesity risk [22,23]. In contrast, a cohort study in Brazil indicated that there was no significant association between birth weight and obesity and a weak but significant association between a high birth weight and overweight among boys [17]. However, these findings regarding the association between birth weight and childhood overweight or obesity have been inconsistent. Also, there are few studies to quantify the relationship between birth weight and BMI levels, especially using some intuitive methods such as restricted cubic splines (RCSs) based on the children and adolescents of all of China. Therefore, the present study aims to investigate and quantify the relationship between birth weight and childhood obesity in China using data from The China National Nutrition and Health Surveillance of Children and Lactating Mothers in 2016–2017.

## 2. Materials and Methods

### 2.1. Study Design and Participants

**Figure 1:**
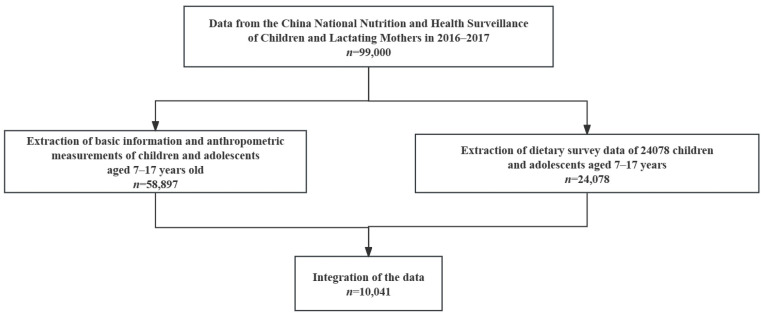
Flowchart for the selection of the study population.

Data were obtained from the China National Nutrition and Health Surveillance of Children and Lactating Mothers in 2016–2017. A representative sample of 31 provinces, autonomous regions, and municipalities was collected using a complex, multi-stage, stratified cluster random sampling method. First, a total of 275 survey sites were randomly selected. Second, two townships/streets were randomly selected from each survey site. Third, two communities/villages were randomly selected from each township/street. A minimum of 280 children and adolescents, with equal numbers of males and females, were selected from schools in each survey site. More details of the study design were explained in an earlier publication [24]. Firstly, we selected 58,897 children and adolescents aged 7–17 years old and collected their basic information and anthropometric measurements. Secondly, we selected 24,078 children and adolescents aged 7–17 years old and collected dietary survey data. Finally, we combined the data from the two aspects above, and 10,041 participants aged 7–17 years were included in this study (Figure 1).

### 2.2. Ethics Approval and Consent to Participate

This study was approved by the ethical committee of the National Institute for Nutrition and Health of the Chinese Center for Disease Control and Prevention. The ethical approval number was 201614. All participants provided informed consent before the study, either themselves or via their guardian.

### 2.3. Data Collection and Measurements

Epidemiological information, such as age, sex, living area, physical activity, income, and parental factors were collected through face-to-face interviews using standardized questionnaires provided by the China Center for Disease Control and Prevention (the China CDC). The anthropometric measurements include birth weight, birth length, height, and weight. Birth weight and length were measured by prescribed standards and requirements in a maternity hospital or a hospital specializing in obstetrics and gynecology. Height was measured with the participants standing upright on the pedal (TZG). Weight measurements were taken using electric scales (G&G tc-200 k, Shanghai, China) with an empty stomach. The anthropometric measurements and questionnaires were collected by professionally trained investigators.

### 2.4. Covariates

To assess the age-related associations of birth weight and overweight or obesity, all subjects were categorized into three age groups reflecting pre-pubertal, pubertal, and post-pubertal stages (7–10, 11–13, and 14–17 for girls; 7–11, 12–14, and 15–17 for boys), according to the Chinese classifications [25]. Birth length was defined as low (birth length < 48.2 cm for boys, birth length < 47.7 cm for girls), normal (birth length = 48.2–52.8 cm for boys, birth length = 47.7–52.0 cm for girls), or high (birth length > 52.8 cm for boys, birth length > 52.0 cm for girls). Physical activity was defined as low level (0–3 days/week), high level (≥4 days/week), or unknown [26]. According to time spent with electronics and watching videos, participants were divided into two groups (<2 h, ≥2 h). The residence area was categorized into urban and rural areas. Engel’s coefficient was calculated as the proportion of total food expenditure to total personal consumption expenditure. We classified the households into six categories (<30%, 30–39%,40–49%,50–59%, ≥60%, and unknown, respectively), according to the recommendation by the Food and Agriculture Organization of the United Nations. The lower the Engel’s coefficient, the better the family economic condition. The nutritional status of each subject was classified as normal, overweight, or obese according to the sex- and age-specific BMI cut-off values for Chinese school-age children and adolescents. Subjects were divided into six categories by birth weight (by 500 g intervals of birth weight from 2500 to 4499 g; birth weights below 2500 were grouped into one category, and birth weights above 4500 were grouped into one category: group A (<2500 g), group B (2500–2999 g), group C (3000–3499 g), group D (3500–3999 g), group E (4000–4499 g), and group F (≥4500 g). The birth weight category of 3000 to 3499 g was chosen as the reference group, containing the largest number of children.

### 2.5. Statistical Analysis

All statistical analysis was conducted with SAS version 9.4, and the plot in this study was made using R version 4.2.2. Median and interquartile range (IQR) were examined for continuous variable given non-normality. The continuous variable was compared using the Kruskal–Wallis rank-sum test between subgroups. The categorical data were expressed as frequencies and percentages and they were compared using *χ*^2^ tests. To eliminate the impact of other factors on the associations, logistic regression analyses were used to assess the strength of the associations between birth weight and classified obesity-related outcomes (overweight and obesity) at 7 to 17 years old in four multivariable-adjusted models: Model 1 was a crude model; Model 2 was adjusted for age, sex, birth length, and residence area; Model 3 was adjusted for variables in Model 2, as well as total energy intake, physical activity, and electronic/video time; Model 4 was adjusted for the variables in Model 3, as well as Engel’s coefficient, parental hypertension, parental diabetes, and parental education level. All the covariables were transformed into dummy variables before conducting the adjustive process. The odds ratios (ORs) and 95% confidence intervals (CIs) were presented. Changes in the effect of birth weight on overweight and obesity with age were analyzed. Also, restricted cubic spline (RCS) in logistic regression procedures was used to test whether there was a dose–response association for birth weight as a continuous variable with the risk of childhood overweight or obesity. Two-tailed tests were used, and the results were considered significant at 0.05.

## 3. Results

### 3.1. Basic Characteristics of the Research Population

A total of 10,041 participants were included in the study, including 5025 boys and 5016 girls. There was an equal distribution of boys and girls. The basic characteristics of the subjects stratified by birth weight are listed in Table 1. There was statistically significant difference for sex, birth length, residence area, Engel’s coefficient, parental education level, and nutritional status among birth weight groups. The numbers (percentages) of overweight in group A to F were 24 (9.1%), 145 (9.9%), 505 (10.5%), 385 (14.2%), 106 (17.1%), and 19 (12.8%), respectively. The numbers (percentages) of obesity in group A to F were 16 (6.1%), 88 (6.0%), 404 (8.4%), 312 (11.5%), 99 (16.0%), and 26 (17.5%), respectively. For all participants, 11.8% were overweight, 9.4% were obese, and 69.8% were normal.

### 3.2. The Relationship between Birth Weight and the Risk of Overweight or Obesity

**Figure 2:**
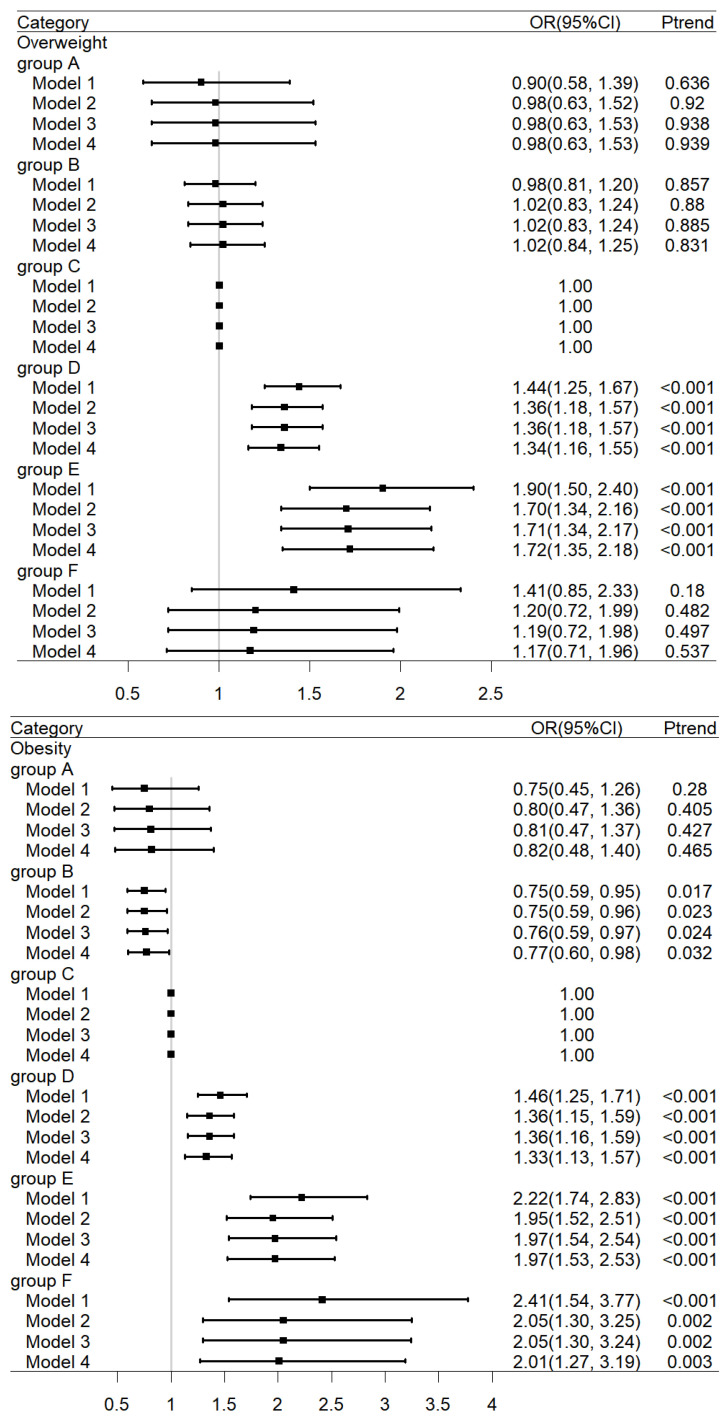
Associations between birth weight and the risk of overweight or obesity. The birth weight category of 3000 to 3499 g was chosen as the reference group. Model 1 was a crude model; model 2 was adjusted for age, sex, birth length and residence area; model 3 was adjusted for variables in model 2, as well as total energy intake, physical activity, and electronic video time; model 4 was adjusted for variables in model 3, as well as Engel’s coefficient, parental hypertension, parental diabetes, and parental education level.

Figure 2 shows the relationship between birth weight and weight-related outcomes (overweight and obesity), which was obtained using adjusted models with ORs (95%CIs). The birth weight category of 3000 to 3499 g was chosen as the reference group, containing the largest number of children. Compared with group C, the multivariable-adjusted ORs (Model 4) for overweight were 1.02 (95%CI 0.84, 1.25), 1.34 (95%CI 1.16, 1.55), 1.72 (95%CI 1.35, 2.18), and 1.17 (95%CI 0.71, 1.96) in group B to F, respectively. Compared with the group C, the multivariable-adjusted ORs (Model 4) for obesity were 0.77 (95%CI 0.60, 0.98), 1.33 (95%CI 1.13, 1.57), 1.97 (95%CI 1.53, 2.53), and 2.01 (95%CI 1.27, 3.19) in group B to F, respectively. Overall, children in group B were less likely to be obese, whereas children in groups D and E were more vulnerable to overweight and obese.

### 3.3. The Relationship between Birth Weight and the Risk of Overweight or Obesity with Age

Table 2 shows the relationship between birth weight and weight-related outcomes (overweight and obesity), which was obtained using adjusted model 4. For the pre-pubertal stage, compared with the group C, the multivariable-adjusted ORs for overweight were 1.89 (95%CI 1.34, 2.66) and 1.86 (95%CI 1.33, 2.61) for obesity in group E. For the pubertal stage, compared with group C, the multivariable-adjusted ORs for overweight were 1.53 (95%CI 1.17, 2.01) and 1.43 (95%CI 1.04, 1.99) for obesity in group D; the multivariable-adjusted ORs for obesity were 1.82 (95%CI 1.09, 3.07) in group E. For the post-pubertal stage, compared with group C, the multivariable-adjusted ORs for overweight were 1.46 (95%CI 1.10, 1.96) and 1.69 (95%CI 1.41, 2.50) for obesity in group D; the multivariable-adjusted ORs for overweight were 2.00 (95%CI 1.23, 3.27) and 3.18 (95%CI 1.75, 5.80) for obesity in group E. After stratifying by age, the relationships between birth weight and children’s overweight or obesity were not significant in group A and group B. Children in groups D and E were at greater risk of developing obesity as their age increased in the pubertal and post-pubertal stage. Moreover, children in the post-pubertal stage had a slightly higher risk of overweight and obesity than those in the pre-pubertal and pubertal stage.

### 3.4. The Relationship between Birth Weight as a Continuous Variable and the Risk of Being Overweight and Obese

To show the relationship visually, odds ratio (OR) curves derived from restricted cubic splines were used to test the dose–response association of birth weight as a continuous variable with the risk of childhood overweight and obesity, and four knots at prespecified locations was applied according to the percentiles of the distribution of birth weight (5%, 35%, 65%, and 95%).

**Figure 3:**
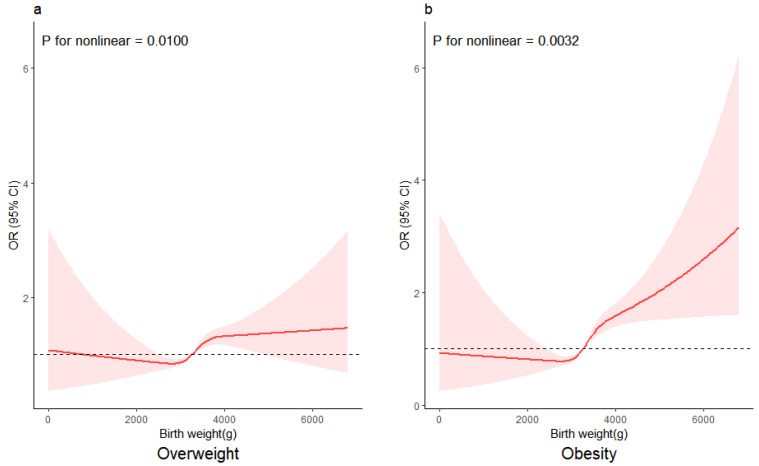
Nonlinear associations between birth weight and classified obesity-related outcomes (overweight and obesity) risk. The associations were examined via multivariate logistic regression models based on restricted cubic splines. (**a**) Birth weight and the risk of being overweight. (**b**) Birth weight and the risk of obesity. The red lines represent estimates of odds ratios, and shaded areas represent a 95% confidence interval (95%CI). The dashed line represents OR=1.

Figure 3 shows nonlinear associations of the full range of birth weight with the odds of childhood overweight and obesity in children after adjustment for confounding factors. In Figure 3a, inverse associations of birth weight with overweight were observed for birth weights below 2778.9 g. Participants with a birth weight exceeding 3253.8 g seemed to have a higher risk of being overweight as their birth weight increased (*p* for nonlinearity = 0.0100). Figure 3b shows that the OR for obesity decreased as birth weight increased to approximately 2733.7 g. Children with a birth weight above 3263.3 g appeared to have a higher risk of obesity as their birth weight increased (*p* for nonlinearity = 0.0032).

### 3.5. The Relationship between Birth Weight and the Risk of Overweight and Obesity by Gender

**Figure 4:**
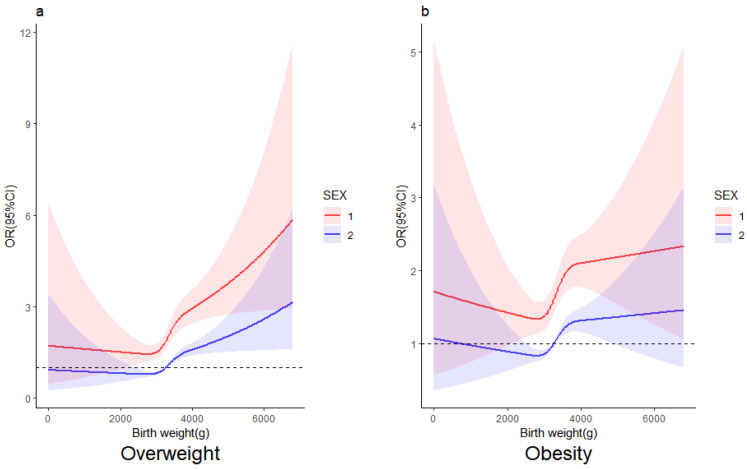
Nonlinear associations between birth weight and classified obesity-related outcomes (overweight and obesity) risk by gender. The associations were examined via multivariate logistic regression model 2 based on restricted cubic splines based on the AIC minimum. (**a**) Birth weight and the risk of being overweight. (**b**) Birth weight and the risk of obesity. The solid lines represent estimates of odds ratios and shaded areas represent a 95% confidence interval (95%CI). Red line for boys, blue line for girls. The dashed line represents OR = 1.

Figure 4 shows the relationship between birth weight and the risk of overweight and obesity by gender, respectively. As shown in Figure 4, there were roughly the same trends in the relationship between birth weight and childhood obesity among boys and girls aged 7 to 17. However, it can be observed that the risks of overweight and obesity are stronger in boys.

## 4. Discussion

In the present study, the results showed that the lower part of normal birth weight (2500–2999 g) was associated with a lower risk of overweight or obesity, and a positive association between higher birth weight and overweight or obesity was observed. However, the association between a low birth weight (<2500 g) and overweight or obesity was not statistically significant.

Our findings are consistent with previous studies [13,27,28]. Two prospective cohort studies of Chinese adults, Shanghai Women’s Health Study and Shanghai Men’s Health Study, examined non-linear associations between the full range of birth weight and risk of obesity. A higher birth weight (>3200 g) was related to a higher prevalence of obesity and a lower birth weight (2500–3100 g) was related to a lower obesity rate [27]. A study of 22 countries in the WHO European Region found that a higher birth weight (>4000 g) was associated with a higher risk of being overweight [28]. The results of a Shanghai twin cohort study of 3393 pairs found that twins with a higher birth weight (≥3000 g) had a higher rate of overweight and obesity [13]. A possible reason for this may be higher levels of growth factors due to a higher birth weight [29,30]. For instance, insulin and insulin-like growth factors I and -II may increase the risk of obesity in children and adults. Maternal overnutrition could increase the risk of obesity in fetal life, and this would track into later life. Intrauterine nutrition is associated with birth weight, and birth weight makes a big difference in the development of children’s obesity [31,32,33,34]. These findings also confirmed the Developmental Origins of Health and Disease (DOHaD) hypothesis that, in addition to lifestyle and genetic inheritance in adulthood, early life environmental factors, including nutrition, influence the risk of developing certain adult NCDS [35].

Nevertheless, some studies were partially inconsistent with our results. Two systematic reviews came to similar results with a large amount of subjects from Asia and China: a high birth weight (>4000 g) was associated with increased obesity risk, while a low birth weight (<2500 g) was associated with a decreased risk of obesity. A cross-sectional study of 16,580 subjects aged 7 to 17 in six Chinese cities indicated J-shaped relationships between birth weight and BMI. A higher birth weight (≥3500 g) increased the risk of childhood overweight and obesity, and lower birth weights (1500–2999 g) decreased the risk of overweight and obesity [36]. However, our research found that children with a low birth weight (<2500 g) showed no significant difference from children with normal birth weight (3000–3499 g) in terms of the risk of overweight and obesity. These inconsistent findings may be due to different age ranges, different target populations, and various definitions of obesity.

Odds ratios were calculated for children’s obesity-related outcomes at different stages of adolescence. We found that children with higher birth weights were more vulnerable to overweight or obesity in the post-pubertal stage. A previous study found a strong association between obesity risk in childhood and adolescence and obesity in adulthood. Participants who were obese in childhood were 12.3 times more likely to be obese in adulthood. If they were obese in adolescence, then they were 45.1 times more likely to be obese in adulthood. For those who are obese in childhood and in adolescence, 55% and 78% are obese in adulthood, respectively [37]. A study in the United States found that children who were overweight at preschool or elementary school were over 5 times more likely to be overweight at the age 12. Other studies have reported similar results [32]. In addition, a cohort survey in southern Brazil found that the mean BMI decreased with age in childhood but increased again during adolescence [38]. Associations of early childhood influences with obesity in later life have, again, confirmed the DOHaD hypothesis. Consequently, birth weight may affect obesity in adolescence and even in adulthood. Obesity in childhood and adolescence is a strong predictor of obesity and other adverse health consequences in adulthood.

Sex differences have been reported in several studies from developed and developing countries [39,40,41]. A double-blind cluster-randomized controlled study in children aged between 7 and 10 years found that a higher birth weight (>3100 g) could increase the risk of being overweight and obese for boys but not for girls, compared to the reference group (2900–3100 g) [39]. Nonetheless, a secondary analysis of an Australian nationally representative cross-sectional study found that a high birth weight (>4000 g) was related to a higher risk of childhood overweight and obesity among boys and girls aged 4 to 5, whereas a low birth weight (<2500 g) was associated with a lower risk of childhood overweight and obesity in girls but not in boys [40]. Another cross-sectional study of children aged between 7 and 10 years conducted in 12 countries revealed that a U-shaped association between birth weight and the risk of childhood obesity was observed in boys, whereas a positive association was found in girls [41]. The results from a Shanghai twin cohort of 3393 pairs found that male twins had a much higher rate of overweight and obesity than female twins [13]. Our research found that the associations between birth weight and overweight or obesity were stronger among boys. These are consistent with An Pan’s results, which concluded that boys had a higher prevalence of overweight and obesity than girls among children and adolescents in China [42]. Given the growth in later life, this may be due to the differences in body composition and the cultural preference for boys in terms of food allocation and caretaking [40,43].

The advantages of this study include a relatively large and representative sample size including children from all over China. However, several limitations should be acknowledged. First, the cross-sectional study precludes us from making cause-and-effect inferences. Second, as the information on maternal BMI, gestational age, and breastfeeding situation were not collected, we may not be able to fully control for the effect of this variable on the association of birth weight with the risk of childhood obesity. Finally, we were unable to obtain indicators other than height and weight, such as hip circumference, due to our limited data. Also, BMI is not sufficient as the only means of classifying a person as overweight or obese. However, BMI allows comparison between children of the same sex and age. And that is exactly what our study did. Therefore, the collection of indicators other than height and weight should be emphasized.

## 5. Conclusions

In summary, this study examined the relationship between birth weight and overweight and obesity. A higher birth weight increased the risk of childhood overweight and obesity, and lower birth weights decreased these risks. These findings provide a new insight into preventing overweight and obesity in children and adolescents.

## Figures and Tables

**Table 1:** Basic characteristics of the study participants.

	Total	Birth Weight, *n* (%)						*p*
Characteristics		A	B	C	D	E	F	
Participants, *n* (%)	10,041 (100.0)	263 (2.6)	1466 (14.6)	4824 (48.0)	2720 (27.1)	619 (6.2)	149 (1.5)	
Sex, *n* (%)								<0.001
boys	5025 (50.0)	108 (41.1)	629 (42.9)	2325 (48.2)	1504 (55.3)	367 (59.3)	92 (61.7)	
Age group, %								0.166
pre-pubertal	4833 (48.1)	131 (49.8)	739 (50.4)	2265 (47.0)	1328 (48.8)	308 (49.8)	62 (41.6)	
pubertal	2772 (27.6)	68 (25.9)	406 (27.7)	1371 (28.4)	714 (26.3)	165 (26.7)	48 (32.2)	
post-pubertal	2436 (24.3)	64 (24.3)	321 (21.9)	1188 (24.6)	678 (24.9)	146 (23.6)	39 (26.2)	
Birth length, %								<0.001
low	1728 (17.2)	151 (57.4)	375 (25.6)	712 (14.8)	377 (13.9)	79 (12.8)	34 (22.8)	
normal	7332 (73.0)	107 (40.7)	1018 (69.4)	3731 (77.3)	2017 (74.2)	384 (62.0)	75 (50.3)	
high	981 (9.8)	5 (1.9)	73 (5.0)	381 (7.9)	326 (12.0)	156 (25.2)	40 (26.9)	
Nutritional status, %								<0.001
underweight	906 (9.0)	39 (14.8)	214 (14.6)	430 (8.9)	183 (6.7)	29 (4.7)	11 (7.4)	
normal	7006 (69.8)	184 (70.0)	1019 (69.5)	3485 (72.2)	1840 (67.7)	385 (62.2)	93 (62.4)	
overweight	1184 (11.8)	24 (9.1)	145 (9.9)	505 (10.5)	385 (14.2)	106 (17.1)	19 (12.8)	
obesity	945 (9.4)	16 (6.1)	88 (6.0)	404 (8.4)	312 (11.5)	99 (16.0)	26 (17.5)	
Residence area, %								<0.001
urban	4953 (49.3)	122 (46.4)	713 (48.6)	2218 (46.0)	1448 (53.2)	352 (56.9)	100 (67.1)	
rural	5088 (50.7)	141 (53.6)	753 (51.4)	2606 (54.0)	1272 (46.8)	267 (43.1)	49 (32.9)	
Total energy, kcal/d	1827.4 (884.2)	1810.4 (781.9)	1800.0 (883.6)	1817.2 (882.6)	1835.2 (904.2)	1883.1 (923.9)	1911.9 (781.6)	0.078
Physical activity, %								0.573
low	3483 (34.7)	98 (37.3)	499 (34.0)	1641 (34.0)	976 (35.9)	216 (34.9)	53 (35.6)	
high	4356 (43.4)	109 (41.4)	639 (43.6)	2101 (43.6)	1159 (42.6)	287 (46.4)	61 (40.9)	
unknown	2202 (21.9)	56 (21.3)	328 (22.4)	1082 (22.4)	585 (21.5)	116 (18.7)	35 (23.5)	
Electronic/video time, %								0.424
<2 h	6984 (69.6)	173 (65.8)	1025 (69.9)	3389 (70.3)	1878 (69.0)	416 (67.2)	103 (69.1)	
≥2 h	3057 (30.5)	90 (34.2)	441 (30.1)	1435 (29.8)	842 (31.0)	203 (32.8)	46 (30.9)	
Engel’s coefficient, %								<0.001
≥60%	203 (2.0)	4 (1.5)	29 (2.0)	98 (2.0)	63 (2.3)	8 (1.3)	1 (0.7)	
50–59%	277 (2.8)	4 (1.5)	52 (3.6)	133 (2.8)	72 (2.7)	14 (2.3)	2 (1.3)	
40–49%	272 (2.7)	5 (1.9)	53 (3.6)	133 (2.8)	65 (2.4)	15 (2.4)	1 (0.7)	
30–39%	686 (6.8)	12 (4.6)	109 (7.4)	353 (7.3)	171 (6.3)	38 (6.1)	3 (2.0)	
<30%	1710 (17.0)	33 (12.6)	219 (14.9)	878 (18.2)	464 (17.1)	91 (14.7)	25 (16.8)	
unknown	6893 (68.7)	205 (78.0)	1004 (68.5)	3229 (66.9)	1885 (69.3)	453 (73.2)	117 (78.5)	

The median and interquartile range (IQR) were examined for continuous variables given non-normality. The categorical data are expressed as frequencies and percentages.

**Table 2:** Associations between birth weight and the risk of overweight and obesity with age.

Group	Pre-Pubertal Stage		Pubertal Stage		Post-Pubertal Stage	
	OR (95%CI)	*p*	OR (95%CI)	*p*	OR (95%CI)	*p*
A						
Overweight	1.05 (0.55–1.98)	0.890	0.59 (0.23–1.54)	0.282	1.28 (0.55–2.97)	0.574
Obesity	0.74 (0.36–1.53)	0.419	0.40 (0.09–1.69)	0.212	2.42 (0.88–6.67)	0.087
B						
Overweight	1.00 (0.75–1.33)	0.981	1.00 (0.68–1.45)	0.984	1.03 (0.68–1.56)	0.894
Obesity	0.73 (0.54–1.01)	0.057	0.93 (0.58–1.49)	0.764	0.64 (0.31–1.34)	0.238
C						
Overweight	reference		reference		reference	
Obesity	reference		reference		reference	
D						
Overweight	1.16 (0.93–1.45)	0.182	1.53 (1.17–2.01)	0.002	1.46 (1.10–1.96)	0.010
Obesity	1.18 (0.95–1.46)	0.135	1.43 (1.04–1.99)	0.030	1.69 (1.14–2.50)	0.009
E						
Overweight	1.89 (1.34–2.66)	<0.001	1.33 (0.82–2.16)	0.245	2.00 (1.23–3.27)	0.006
Obesity	1.86 (1.33–2.61)	<0.001	1.82 (1.09–3.07)	0.023	3.18 (1.75–5.80)	<0.001
F						
Overweight	1.35 (0.63–2.88)	0.444	1.01 (0.40–2.53)	0.991	1.00 (0.34–3.00)	0.994
Obesity	1.85 (0.96–3.57)	0.066	2.29 (0.99–5.31)	0.052	2.97 (0.96–9.18)	0.059

## Data Availability

The data are not publicly available according to the policy of the National Institute for Nutrition and Health, Chinese Center for Disease Control and Prevention.
